# Medical Care in a Free Clinic: A Comprehensive Evaluation of Patient Experience, Incentives, and Barriers to Optimal Medical Care with Consideration of a Facility Fee

**DOI:** 10.7759/cureus.500

**Published:** 2016-02-19

**Authors:** Antoinette Birs, Xinwei Liu, Bee Nash, Sara Sullivan, Stephanie Garris, Marvin Hardy, Michael Lee, Judith Simms-Cendan, Magdalena Pasarica

**Affiliations:** 1 University of Central Florida College of Medicine; 2 FIRE Module, University of Central Florida College of Medicine; 3 Consulting Department, Sullivan Evaluation Services; 4 Grace Medical Home; 5 Medical Director, Grace Medical Home; 6 Pharmacology, University of Central Florida College of Medicine; 7 Medical Education, University of Central Florida College of Medicine

**Keywords:** free clinic, facility fee, barriers to healthcare, uninsured, healthcare improvement

## Abstract

Free and charitable clinics are important contributors to the health of the United States population. Recently, funding for these clinics has been declining, and it is, therefore, useful to identify what qualities patients value the most in clinics in an effort to allocate funding wisely. In order to identify targets and incentives for improvement of patients’ health, we performed a comprehensive analysis of patients’ experience at a free clinic by analyzing a patient survey (N=94). The survey also assessed patient opinions of a small facility fee, which could be used to offset the decrease in funds. Interestingly, our patients believed it is appropriate to be charged a facility fee (78%) because it increases involvement in their care (*r *= 0.69, *p *< 0.001) and self-respect (*r *= 0.66, *p *< 0.001). Incentives to medical care include continuity of care, faith-based care, having a patient medical provider partnership, and charging a facility fee. Barriers include affordable housing, transportation, medication, and accessible information. In order to improve medical care in the uninsured population, our study suggested that we need to: 1) offer continuity of medical care; 2) offer affordable preventive health screenings; 3) support affordable transportation, housing, and medications; and 4) consider including a facility fee.

## Introduction

With the advent of the Affordable Care Act (ACA), Medicaid services have expanded to include: (1) adults under the age of 65 who earn below 138% of the federal poverty line and (2) working families who earn between 100% and 400% of the federal poverty line. These individuals will be provided with tax credits to help them purchase health insurance in the newly created health care marketplace [1]. Currently, not all states have chosen to adopt these new laws. Twenty states have refused to expand Medicaid (as of September 2015), leaving an estimated three million Americans without coverage [2]. Incomplete state participation in the Medicaid expansion has created a healthcare "gap", which includes Americans who are either eligible for Medicaid, but reside in states not expanding the Medicaid program or have an income too high to qualify for Medicaid, but still cannot afford medical insurance despite public subsidies and tax credits.

Free and charitable clinics across the U.S. help bridge this gap in health care coverage and provide services to fit the medical needs of these uninsured Americans. According to the National Association of Free and Charitable Clinics (NAFCC), there are approximately 1,200 free and charitable clinics nationwide, all of which are 501(c)(3) tax-exempt organizations that do not receive funding from the federal government [3]. With an average of over 4,000 patient visits and almost 800 new patients per year, a recent study surveying over 360 clinics nationwide found that there is an increasing demand and need for free and charitable clinics [2, 4]. Without clinics, the uninsured population will not have access to the same standard of medical care and preventive services provided to the insured population and may likely experience a delay in disease diagnosis as is described in a study by Ayanian and colleagues who surveyed over 200,000 Americans [5]. A delay in diagnosis and preventative care can ultimately lead to negative health outcomes and higher healthcare costs for this population [6-7]. It is estimated that patients who have been without insurance for over one year will pay approximately one-fifth of their care out of pocket and typically pay higher fees than the insured [8-9]. Moreover, financial stressors have been shown to lead to increased levels of depression and anxiety [10] and can negatively impact or worsen other mental and physical ailments [11]. These clinics have become a vital contributor to the medical and preventative care of the uninsured [12].

Today, most uninsured patients report that they would either not seek medical care or would use the emergency department if free clinics were not available [4]. Free clinics lessen the burden placed on emergency departments while providing care that is comparable to the national standard of care [13-14]. Clinics are reported to have increased staff friendliness and a generalized positive perception of the depth of medical explanation received and the amount of time spent with the medical provider [15-19]. 

Despite their critical role in the medical care for uninsured or underinsured patients, the NAFCC reports that clinics have suffered an overall 20% decrease in funding [2]. This decrease in funding increases the already existing financial deficit faced by these clinics. One solution may be found in instituting a small facility fee, under the premise that the fee will not negatively affect patient care or patient utilization of free clinics. It has been found that patients are more willing to seek medical care from institutions accepting Medicaid/Medicare where they are expected to pay a sliding scale fee versus facilities where only private insurance is accepted [19]. Studies have demonstrated that improvement in patient care is highly dependent on patient engagement and patient return for the recommended office visits [20]. There is a paucity of data validating the reception and effectiveness of a facility fee, and our study aims to delineate whether a fee will serve as an incentive or barrier to medical care. Our goal is to identify targets and incentives to optimize primary medical care in our clinic by performing a comprehensive evaluation of patient’s experience, concerns, outcomes, and perceptions in a free clinic that may be applied to clinics across the United States.

## Materials and methods

### Study description

This is a retrospective secondary analysis of a patient survey administered as part of an internal periodic evaluation of patient satisfaction. Patients were recruited between September 1-30, 2014 while receiving services at Grace Medical Home, a free clinic in Orlando, Florida. Participation was voluntary. The nature and objectives of the study were explained and informed consent was obtained. To be eligible to complete the survey, participants were required to meet the following requirements: (1) they must be patients of the clinic and (2) be 18 years of age or older. If the patient was a minor, a parent was encouraged to fill out the survey on their behalf. The survey was anonymous and was offered in both English and Spanish. A total of 94 surveys were collected after being completed fully.

The clinic offers adult and pediatric medical services in both primary care and specialty care. The clinic charges a small facility fee ($5, if patients can afford it) for the purpose of meeting the patient's health care needs while taking into account their ability to pay the fee. This fee is meant to offset some of the fixed operating services, such as rent and utilities, and makes up less than 5% of the clinic budget. No patient will be denied medical care if they cannot pay the fee. There is no fee for clinical re-checks, preventive screenings, laboratory assessments, X-rays, sample medications, office visits, or care coordination.

The anonymous survey contains 36 items which collected information regarding: (1) demographic characteristics; (2) options for medical care before joining the clinic; (3) reasons for not having health insurance; (4) reasons for wanting to be a patient of the clinic; (5) health status, health worries, missed work/school days, information availability before and after joining the clinic; (6) reasons for missing appointments and not taking prescribed medicine; (7) issues with transportation to clinic; (8) experience of being a patient in the clinic; and 9) experience with and attitude towards the facility fee.

Certain patient demographics were not collected; however, the clinic reports that they serve a diverse race, sex, and age group. Patients are uninsured residents of Orange County, Florida who are at or below 200% of the federal poverty level.

The retrospective analysis presented in this study was reviewed and approved by the Institutional Review Board at the University of Central Florida (approval #SBE-14-10831).

### Statistical analysis

Patient characteristics are represented by descriptive statistics (frequencies and valid percentages). Pearson Chi-square or the Binomial test was used to determine significant differences in categorical data. To study the relationship between the clinic facility fee and accountability as well as self-respect, correlational analyses were conducted. Only applicable responses were analyzed. All tests were two-sided, and *p*-values < 0.05 were considered statistically significant. Statistical analyses were conducted in IBM SPSS Statistics 22.0 (SPSS Inc, Chicago, IL).

## Results

### Patients’ financial characteristics and access to healthcare

Ninety-four patients answered the survey. Most of the patients participating in this study were unemployed (40.2%), and their living conditions were variable with the majority renting or owning an apartment (68.9%) as can be seen in Table 1. For transportation to the clinic, most used their own car (73.5%). Before joining the free clinic, most patients (37.1%) would not get care, and 30.3% would go to emergency room and urgent care facilities. The clinic's patients did not have health insurance for multiple reasons, including not being able to afford the Affordable Care Act Marketplace or private insurance.

**Table 1:** Patients’ Characteristics and Access to Health Care This table presents patients’ characteristics (as determined by employment status, living situations, and mode of transportation) and also their access to health care (as determined by the self-reported healthcare alternative choice if not Grace Medical Home, knowledge as how to access medical care or where to go for help, and concerns related to public health options). Data were generated from ninety-four (94) surveys. Not all questions were answered by all participants. Data are presented here in frequencies and valid percentages.

Patient Characteristics and Access to Healthcare	Patient Selection: Frequency (Percentage)
Employment Status	
Unemployed	33 (40.2%)
Full-time employment; no benefits	28 (34.1%)
Seasonal work; not a permanent job	5 (6.1%)
Under employed (less than 30 hours/week)	16 (19.5%)
Living Situation	
Rent or own an apartment or house	60 (68.9%)
Staying with friends/relatives	23 (26.7%)
Shelter	0 (0%)
Transitional housing	1 (1.1%)
Staying on the street, in car, in woods, etc.	1 (1.1%)
Motel	2 (2.3%)
Modes of Transportation	
Drive my own car	64 (73.5%)
Borrow car from friend/family member	6 (6.9%)
A friend or family member drives me	12 (13.8%)
Take the bus	8 (9.4%)
Walk or ride bike	3 (3.5%)
Healthcare Alternative (If not Grace Medical Home)	
Health clinic	18 (20.2%)
Doctor’s office	3 (3.4%)
Emergency room	27 (30.3%)
Urgent care clinic	8 (9.0%)
Would not get care	33 (37.1%)
Unsure How to Access Medical Care or Where to Go for Help; Concerns about Enrolling in Public Healthcare Options (Medicaid, Medicare, ACA, etc)	
Don’t know how to enroll	6 (6.4%)
Too complicated or difficult	6 (6.4%)
Won’t have specialist I need	16 (17.0%)
Don’t want insurance	26 (27.7%)
Can’t afford marketplace premium	55 (58.5%)
Can’t afford cost of co-pays	41 (43.6%)
Plans don’t cover benefits I’m looking for	8 (8.5%)
Limited choices	15 (16.0%)

### Patients’ health and financial concerns before joining the free clinic

Before joining the free clinic, a large majority of patients were worried about not knowing how to access medical care and where to go for help (80.5%) as is represented by the data in Table 2. Patients had a variety of medical concerns ranging from morbidities due to chronic diseases to the lack of early detection screenings.

**Table 2:** Patients’ Health and Financial Concerns Before Joining the Free Clinic This table presents patients’ medical and financial concerns before joining Grace Medical home. Data were generated from ninety-four (94) surveys. Not all questions were answered by all participants. Data are presented here in frequencies and valid percentages. *Participants were able to select multiple responses.

Health and Financial Concerns Before Joining the Free Clinic	Number of Responses: Frequency (Percentage)
Medical Concerns	
Overweight	37 (50.0%)
Low energy due to illness/condition	46 (62.2%)
Chest pain/heart palpitations	14 (19.7%)
Preventive cancer/early detection screenings not done	28 (38.9%)
Financial Concerns	
Missing work due to illness	32 (45.7%)
Financial crisis or bankruptcy due to medical issues	40 (52.6%)
Unsure how to access medical care or where to go for help	62 (80.5%)

### Patients’ medical, financial and faith outcomes resulting from receiving medical care from the free clinic

**Figure 1:**
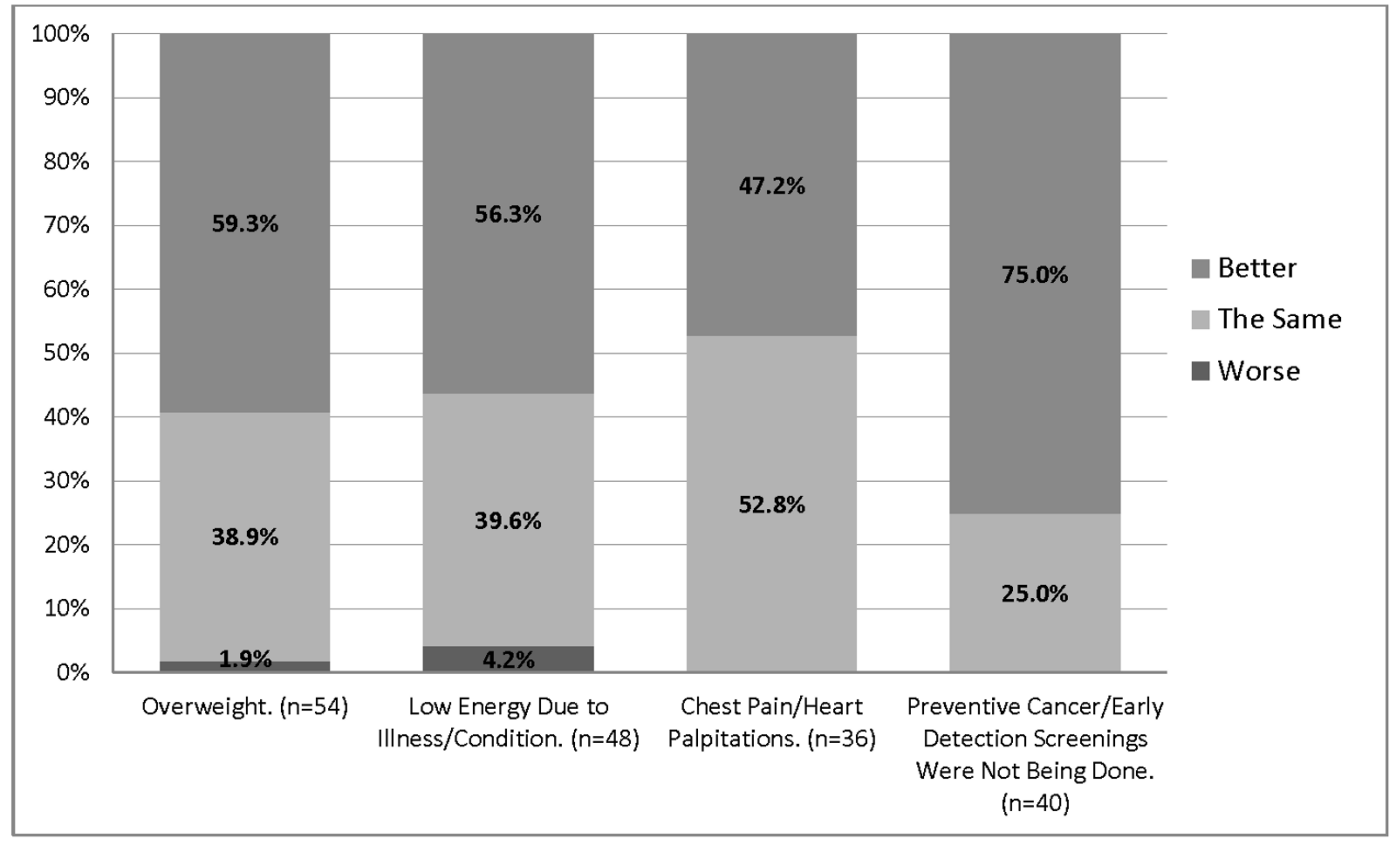
Medical Outcomes of Patients Receiving Medical Care in the Free Clinic This figure presents the medical outcomes of patients as better, the same, or worse since they joined the free clinic. Patients were asked about general health concerns, access to preventative studies and energy levels. Data were generated from ninety-four (94) surveys. Not all questions were answered by all participants (the range was 85-94 responses). Data is presented here in valid percentages. Participants were able to select multiple responses.

**Figure 2:**
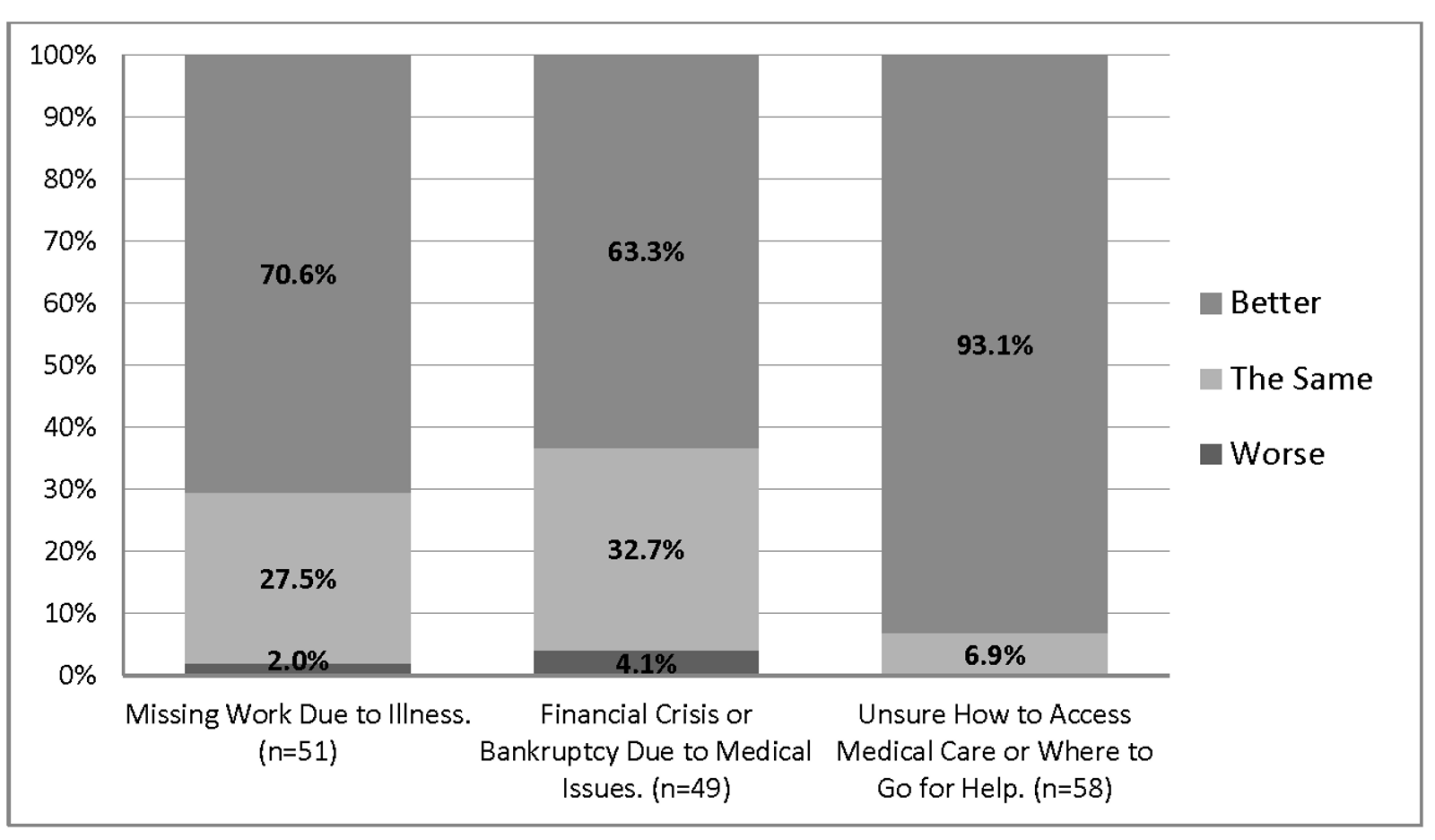
Financial Outcomes of Patients Receiving Medical Care in the Free Clinic This figure presents the financial outcomes of patients as better, the same, or worse since they have joined the free clinic. Patients were asked questions about long term financial outcomes and medical care access. Data were generated from ninety-four (94) surveys. Not all questions were answered by all participants (the range was 85-94 responses). Data is presented here in valid percentages. Participants were able to select multiple responses.

Data is presented in Figures 1-2. Significantly more patients (94.7%, *p *< 0.001) reported that their health had improved since joining the free clinic, compared to 2.2% who either disagreed or felt neutral. Patients with improved health were more likely to feel that they were treated with courtesy and dignity (*r* =0.61, *p *< 0001). Patients reported receiving more prevention and screening services (*p *< 0.05), missing fewer work days due to illnesses (70.6% *p* < 0.05), and had fewer concerns about a financial crisis and bankruptcy due to medical illnesses. Patients also learned how to access medical care and where to go for help.

### Incentives and potential barriers to optimal medical care in the free clinic

Data representing potential barriers for medical care: appointment and treatment noncompliance and paying a fee is presented in Table 3. We found that patients missed their clinic appointments because of various reasons with transportation problems (22.3%), appointment rescheduling problems (18.1%), and inability to get time off from work/school (14.9%) being the most commonly selected.

**Table 3:** Patient Perceptions of Grace Medical Home’s Qualities This table presents qualities most valued in free clinics and qualities of Grace Medical Home. Data were generated from ninety-four (94) surveys. Not all questions were answered by all participants. Data are presented here in frequencies and valid percentages. Participants were able to select multiple responses.

Qualities Most Valued by Patient at Grace Medical Home	Number of Responses: Frequency (Percentage)
Primary care	69 (73.4%)
Specialty care	48 (51.1%)
Affordable medications	47 (50.0%)
Relationships with staff/volunteers	59 (62.8%)
Sets up appt and coordinates referrals	41 (43.6%)

Patients valued primary care (73.4%) and specialty care (51.1%), but also affordable medications (50.0%) and having coordinated appointments and referrals (43.6%).

### Facility fee

**Figure 3:**
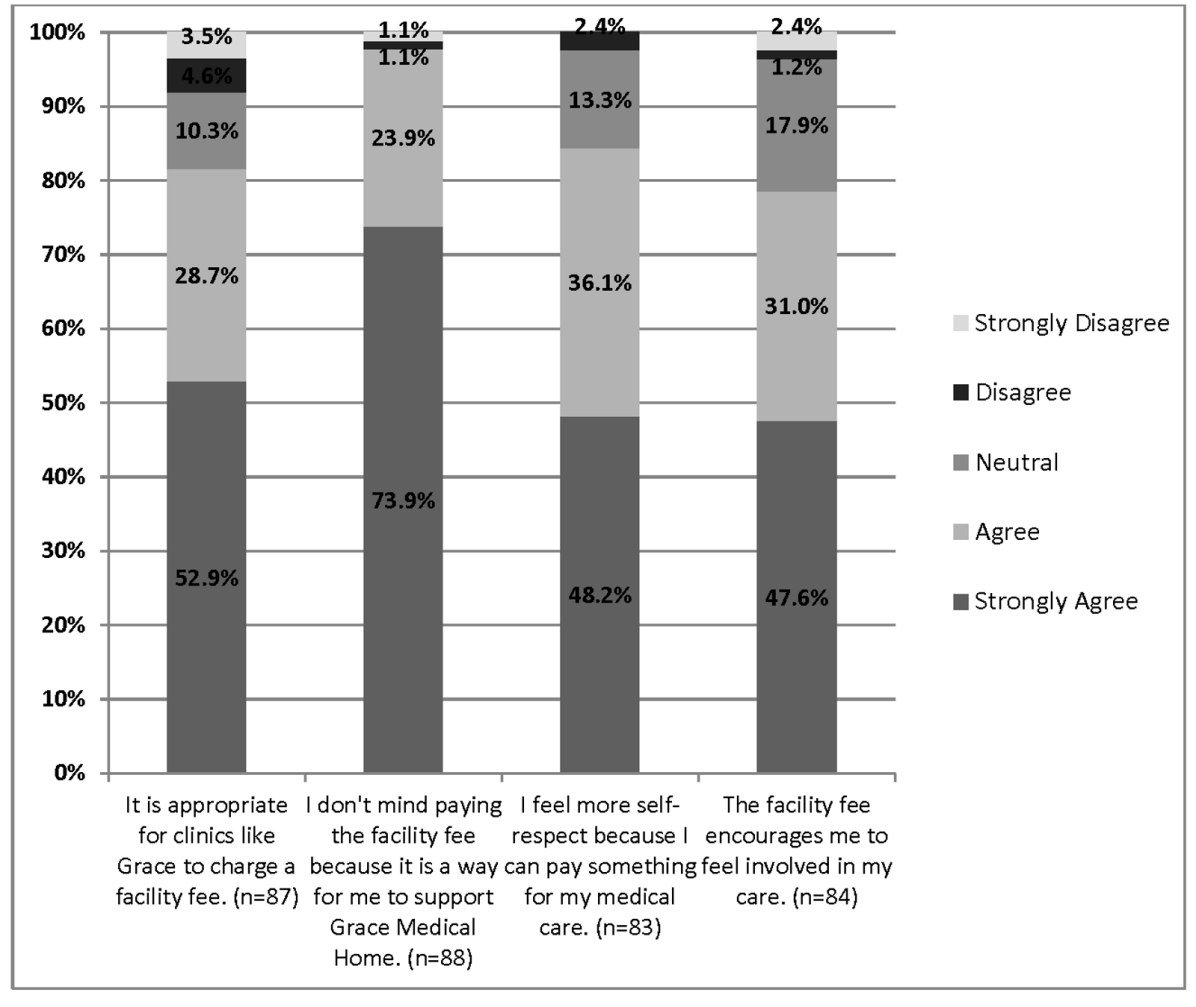
Positive Patients’ Attitude Towards Paying a Facility Fee for Medical Care in the Free Clinic This figure presents the response of positive statements towards paying a facility fee in the free clinic. Data were generated from ninety-four (94) surveys. Not all questions were answered by all participants (the range was 90-94 responses). All responses were on a 1-5 Likert scale. Data is presented here is in valid percentages. Participants were able to select multiple responses.

**Figure 4:**
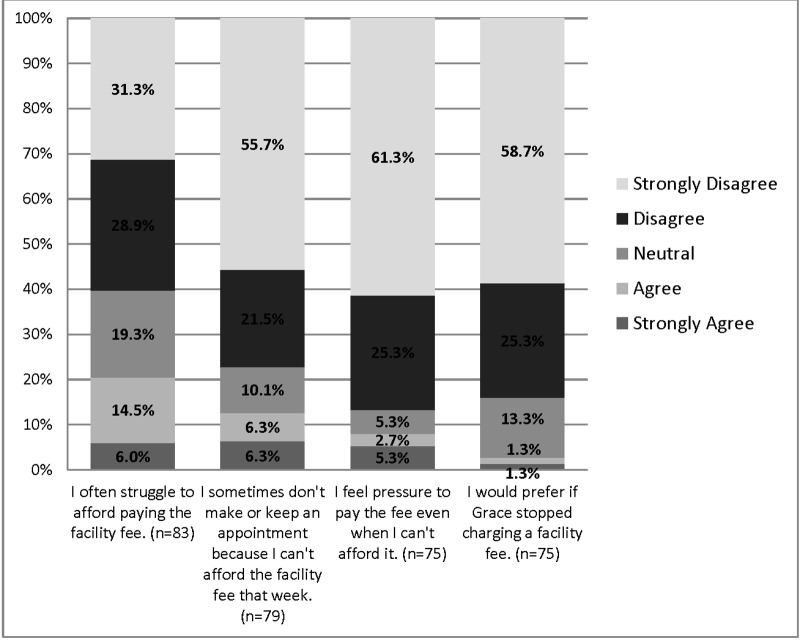
Negative Patients’ Attitude Towards Paying a Facility Fee for Medical Care in the Free Clinic This figure presents the response of negative statements towards paying a facility fee in the free clinic. Data were generated from ninety-four (94) surveys. Not all questions were answered by all participants (the range was 90-94 responses). All responses were on a 1-5 Likert scale. Data is presented here is in valid percentages. Participants were able to select multiple responses.

We then studied patients’ attitudes towards paying a small sliding scale facility fee (data presented in Figures 3-4 and Table 4). Interestingly, significantly more patients agreed or strongly agreed that it is appropriate for the clinic to charge the facility fee compared to those that disagreed (81.6% vs. 8.1%, *p *< 0.001). Significantly more patients felt that by paying a fee they supported the free clinic compared to the ones that disagreed (97.8% vs 2.2%, *p *< 0.001). In turn, paying the facility fee enhanced patients’ level of self-respect (84.3% vs. 2.4%, *p* < 0.001). Further analysis showed that patients who reported that paying the facility fee was appropriate, also reported that paying this facility fee encouraged them to be more involved in their care (*r* = 0.69,  *p* < 0.001) and have more self-respect (*r *= 0.66, *p* < 0.001).

**Table 4:** Correlation Analysis of Patient Willingness to Pay Facility Fee This table presents correlations (*r*-values) between questions related to patients’ attitude towards the facility fee. Correlations which are significant with *p* < 0.01 level are in bold.

Questions	1	2	3	4	5
1. Struggle to pay fee	1				
2. Can’t keep apt due to fee	0.75	1			
3. Fee encourages me to feel involved in care	0.63	0.61	1		
4. More self-respect paying something for care	0.59	0.39	0.52	1	
5. It is appropriate to charge a fee	0.68	0.54	0.69	0.65	1

Only 20.5% of patients reported that they often struggle to pay the facility fee and 12.7% reported that this resulted in missing appointments. A small percentage of patients (8.0%) felt pressured to pay the fee and more patients felt that the clinic should continue the facility fee compared to those who thought it should be discontinued (84.0% vs. 2.7%, *p* <0.001).

Furthermore, even though patients who reported struggling to pay the facility fee were more likely to miss their appointments due to the fee (*r* = 0.68, *p* < 0.001), they also reported that paying the facility fee encouraged them to be more involved in their care (*r *= 0.63, *p* < 0.001), have more self-respect (*r* = 0.59, *p* < 0.001), and ultimately believed that it was appropriate to be charged a fee (*r *= 0.68, *p *< 0.0001).

## Discussion

Previous studies evaluating the medical care in free and charitable clinics have focused on tightly defined parameters, such as patient satisfaction and medical outcomes. While these studies contribute valuable information to our understanding of medical care in free clinics, our study specifically aims to assess incentives or potential barriers to optimal medical care, willingness to contribute a small fee, and if this fee increases patients' perception of ownership over one’s health.

### Patient characteristics

Before joining the free clinic, most patients reported seeking care at the emergency department or urgent care facilities, which typically have a high cost for the medical facility as well as the patient [21]. This practice increases the burden on local emergency departments, turning them into expensive *de facto* primary care facilities. One reason why emergency room visits may be so costly is that a significant portion of the care provided will go uncompensated. The Centers for Medicare and Medicaid Services estimate that about 55% of all emergency services nationwide will not be paid for. This financial burden is redistributed within the hospital, onto the privately insured, and those who self-pay [22]. Previous reports showed that a tremendous amount of funding allotted for health care could be saved just by offering better health insurance, encouraging all workplaces to carry affordable health insurance, or supporting more free and charitable clinics [4]. The Agency for Healthcare Research and Quality (AHRQ) found that the average expenses for all people who had one or more visits to the emergency room in 2009 were $1,318 [23]. More than half of the patients surveyed are concerned about bankruptcy or financial crisis due to medical issues, which may explain why a staggering majority of patients would not seek medical care at all if they were unable to access care from a free clinic. Delays in seeking treatment may not only worsen a patient’s medical outcome, but also increase the cost of medical care when finally seeking treatment.

### Expectations of care

Most of the patients surveyed came to the free clinic for chronic disease management and affordable medications. Approximately half of the patients report that they came to the free clinic because they wanted a physician to see regularly and who could provide them with continuity of care. One-third of the patients had concerns about not receiving preventive health care and screening services prior to joining the free clinic. This is consistent with previous reports showing that the uninsured do not have access to similar prevention and screening as the insured [5]. Health care costs and suffering from an advanced disease may be decreased by offering screening tests, including pap smears, mammograms, and lipid and diabetes screening. 

### Barriers to care

Transportation may be a barrier to care as 26.5% of the patients did not own a car. Clinic locations near bus lines or walking distance to subway stations should be considered when locating new clinics. Other patients would not seek care because they were worried about missing work due to illness, suggesting the importance of offering appointments after working hours.

Previous research supports the fact that medical care in free clinics is of good quality [4, 13-14]. In our study, almost all patients reported that their health and financial concerns had improved after receiving care at the free clinic. Interestingly, patients reported that the care at Grace Medical Home also encouraged their faith, which was one of their most valued experiences. This suggests that offering medical care in a faith-based institution may increase patient satisfaction and compliance.

Medication compliance dwindled because of either cost or side effects, suggesting the importance of patient education about medication and importance of adhering to the regimen. More frequent visits for clinical reassessment and continuity of care will allow physicians to evaluate the side effect profile and ability to tailor the patient's treatment plan. We do acknowledge that some medications may not be available to our population due to high cost. Thus, developing new models whereby new or enlarged patient assistance programs or reduced price plans could be developed is of paramount importance.

We know that patients who are more engaged in their care report better health [20]. Some institutions, including the one studied here, implemented a sliding scale facility fee, which is believed to improve compliance, accountability, and ultimately medical care. This small facility fee may be a potential barrier to patient care; however, there are no studies currently published in the literature addressing this topic. We found that most patients agreed that it is appropriate to pay a sliding scale facility fee and that doing so made them feel more involved in their own medical care. The data demonstrates that by putting a monetary investment into the visit, patients are more likely to be compliant with medications and physician recommendations and feel more responsible for their medical care. Patients reported improvement in their financial concerns and a reduction of missed workdays secondary to illness. If a patient is able to justify a small facility fee in exchange for fewer sick days and involvement in their medical care with increased self-respect, then the facility fee becomes an incentive to improving health. This study can be used in other free and charitable clinics to determine if a facility fee is appropriate. The primary focus is increased patient care and satisfaction, with the facility fee being an incentive adopted to improve patient care.

This study was conducted in a state that has not expanded Medicaid, so our findings may not be applicable to all states. The survey tool used in this analysis was internally developed and has not been validated at this time; therefore, we acknowledge this methodological limitation and encourage usage of a standardized survey that can be applied to all clinics. Future studies should be conducted on a larger scale to include clinics in several geographic locations to substantiate the generalizability of our findings.

## Conclusions

As the financial support for funding free and charitable clinics decreases [2], there is an acute need to identify the obstacles to optimal medical care in an effort to develop targeted strategies to improve care and efficiency in free and charitable clinics. Most importantly, we explored the patient perception of a facility fee, which we believe will not only improve the patient’s sense of responsibility for their health care but also shrink the expanding deficit that clinics are facing. Several facets of the free clinic, such as continuity of medical care, faith-based care, and charging a facility fee, actually improved patient compliance and engagement. This study is significant as it allowed us to draw conclusions that can be used for future development of policies to improve the health of the poor and underserved. Our study suggests that critical factors to improve medical care in the uninsured population would 1) offer continuity of medical care; 2) offer affordable and preventive medicine; 3) support affordable transportation, housing, and medicine; and 4) adoption of a facility fee to bolster patient compliance and ownership of healthcare.
